# Mild Malnutrition in Children Aged 6–24 Months and Related Influencing Factors: Research From an Outpatient Clinic of Pediatric Health Care

**DOI:** 10.1002/pdi3.70058

**Published:** 2026-06-29

**Authors:** Menghua Yin, Qian Li, Rumeng Yu, Mengxuan Zhou, Wenxiu Wu, Li Song

**Affiliations:** ^1^ Department of Child Health Care The Third Affiliated Hospital of Zhengzhou University Zhengzhou China; ^2^ Department of Oncology Shanghai Children's Medical Center Shanghai Jiao Tong University School of Medicine Shanghai China

**Keywords:** infants and young children, influencing factors, mild malnutrition

## Abstract

Mild malnutrition in infants and young children is an early stage of nutritional deficiency characterized by a slight reduction in nutritional reserves and mild impairment of physiological functions but no obvious clinical symptoms or severe growth retardation. Parents often overlook this condition because typical signs are absent. This cross‐sectional study aimed to investigate the current status of mild malnutrition in infants and young children aged 6–24 months attending a pediatric health clinic, analyze its influencing factors, and provide a basis for nutritional health guidance. The study included 879 infants and young children aged 6–24 months who underwent physical examinations at the Outpatient Clinic of Pediatric Health Care, The Third Affiliated Hospital of Zhengzhou University, China, from December 2024 to August 2025. The survey results showed a detection rate of mild malnutrition of 13.88% (122/879). After including statistically significant factors from univariable analysis in a multivariable logistic regression, the following risk factors were identified: food allergies (odds ratio (OR) = 3.117, 95% confidence interval (CI): 1.902–5.108), low maternal education level (OR = 1.969, 95% CI: 1.166–3.325), low birth weight (OR = 1.759, 95% CI: 1.041–2.973), maternal prepregnancy BMI < 18.5 (OR = 2.197, 95% CI: 1.252–3.855), frequent nighttime awakenings (OR = 2.427, 95% CI: 1.481–3.975), and insufficient complementary feeding frequency (OR = 1.550, 95% CI: 1.030–2.332). The detection rate of mild malnutrition in infants and young children aged 6–24 months at pediatric clinics is relatively high, and clinical work should give sufficient attention to this issue. Proactive guidance should be provided to these children to promote healthy growth.

## Graphical abstract

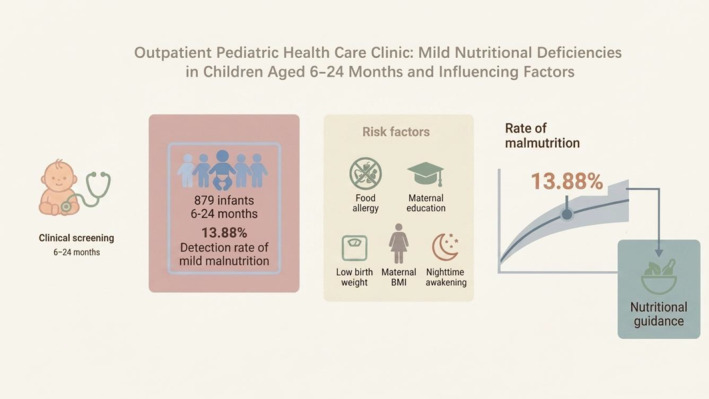

## 1 Introduction

The first 1000 days of life are considered a critical window of opportunity that significantly influences growth and development, as well as lifelong health. Nutrition, one of the most critical environmental factors during this period, directly determines the rate of physical development, strength of immune function, and cognitive potential of infants and young children, and influences the health, educational attainment, income, and birth weight of offspring in adulthood [1, 2] through underlying mechanisms such as gene expression regulation, hormone level programming, and gut microbiota [3]. This imprinting effect of early nutrition has thus significant long‐term impact.

Exclusive breastfeeding for the first 6 months of life is the foundation for ensuring that infants receive optimal nutrition, as the nutrient composition of breast milk is precisely tailored to infants' digestive and absorptive capabilities, comprehensively meeting the needs for growth, immune function, and cognitive development. It plays a central role in early‐life feeding. As infants grow, their demand for nutrients increases substantially. After 6 months of age, breast milk alone can no longer fully meet the infant's growth needs. At this stage, infants' feeding method must transition from exclusive breastfeeding to a family diet, while the care model also requires significant adjustments; caregivers need to acquire scientific knowledge about introducing complementary foods, monitor the infant's feeding responses, and ensure balanced nutrient intake. This dual transformation in feeding methods and care models places high demands on families and caregivers. If issues, such as the delayed introduction of complementary foods, limited variety, nutritional imbalance, or improper feeding, arise during this process, they can easily lead to insufficient or imbalanced nutrient intake in infants and young children. Thus, this stage is a high‐risk period for malnutrition.

With sustained economic growth, advances in medical technology, and the increased awareness of maternal and child health, the incidence of moderate and severe malnutrition among Chinese children has shown a steady annual decline [4]. However, due to the country's large population, the absolute number of malnourished children remains significant. Relevant surveys indicate that 83.9% of malnourished children attending pediatric health clinics have mild malnutrition [5]. Mild malnutrition in infants and young children represents the early stage of nutritional deficiency, often triggered by acute events such as economic hardship or acute illness. It is characterized by unintentional weight loss or a weight gain rate below expectations [6], with slightly reduced body nutrient reserves and mild impairments in physiological functions but no obvious clinical symptoms or severe growth retardation. According to the 2014 Pediatric Nutrition Assessment Criteria established by the Academy of Nutrition and Dietetics (AND) and the American Society for Parenteral and Enteral Nutrition (ASPEN), a diagnosis of mild malnutrition requires meeting any one of the following criteria (while excluding moderate or severe malnutrition): a weight‐for‐height z‐score between −2 and −1, a body mass index (BMI)‐for‐age z‐score between −2 and −1, or a mid‐upper arm circumference (MUAC) z‐score between −2 and −1 [6]. Unlike other diagnostic criteria, this diagnostic system innovatively incorporates the MUAC z‐score as a core diagnostic dimension, building upon traditional growth indicators. The MUAC directly reflects skeletal muscle and subcutaneous fat reserves in a child's upper arm, providing a rapid and objective assessment of the body's protein‐energy nutritional status. MUAC z‐scores are more suitable than traditional absolute MUAC measurements for diagnosing mild‐to‐moderate malnutrition. They accurately track dynamic changes in children's nutritional status and avoid interference from fluid fluctuations and hydration abnormalities in the assessment results, even when children have ascites or edema [7, 8, 9]. This provides a more stable and reliable basis for nutritional diagnosis, aligning with this study's core objectives of the early identification of high‐risk groups and the exploration of targeted influencing factors. Research studies have indicated that mild malnutrition is the most prevalent malnutrition subtype, affecting 8%–21.6% of children in all age groups [10]. Changes in the prevalence of mild malnutrition are more closely and consistently associated with child mortality than changes in the prevalence of severe malnutrition [11]. Within the theoretical framework of public health prevention, addressing and controlling mild malnutrition is the foundational step in establishing a three‐tier prevention system for childhood malnutrition. It is also crucial for effectively reducing the incidence of moderate‐to‐severe malnutrition, holding significant practical implications for improving overall child health. Existing child nutrition research predominantly focuses on moderate‐to‐severe malnutrition, which presents clear health hazards, and established clinical interventions. In contrast, the epidemiological characteristics and core drivers of mild malnutrition remain understudied due to its subtle symptoms and delayed health impacts. This study aimed to address this gap by focusing on the critical developmental window of 6–24 months of age. A systematic analysis of the epidemiological status of mild malnutrition in infants and young children during this period was performed to provide evidence‐based support for the timely detection of developmental deviations, the formulation of scientifically precise nutritional interventions, and their implementation in clinical practice. Ultimately, this study aims to achieve the core objectives of improving nutritional status and promoting the healthy growth of infants and young children aged 6–24 months.

## 2 Population and Methods

### 2.1 Study Population

This was a cross‐sectional study that included infants aged 6–24 months who underwent health examinations at the Outpatient Clinic of Pediatric Health Care, The Third Affiliated Hospital of Zhengzhou University, China, between December 2024 and August 2025. The inclusion criteria were as follows: (1) complete questionnaire and physical examination data and (2) informed consent from family members. The exclusion criteria were as follows: (1) having chronic conditions, such as severe congenital heart disease, chronic gastrointestinal disorders (e.g., Crohn's disease and chronic diarrhea lasting over 1 month), endocrine disorders (e.g., hypothyroidism and growth hormone deficiency), and severe neurological disorders (e.g., cerebral palsy and intellectual disability with dysphagia); (2) having acute conditions, including acute infectious diseases accompanied by fever (temperature ≥ 38.5°C persisting for over 24 hours) or significant diarrhea (≥ 5 episodes of watery stools daily)/vomiting (≥ 3 episodes of non‐feeding‐related physiological vomiting daily) within the past week; and (3) children with any Z‐score (HAZ, WAZ, MUACz) ≤ −2 were excluded to rule out moderate‐to‐severe malnutrition. This study was approved by the Ethics Committee of The Third Affiliated Hospital of Zhengzhou University (2024‐428‐01).

### 2.2 Methods

#### 2.2.1 Physical Measurements

A standardized team of pediatric health workers who were trained and certified through standardized training and assessment, used standardized measurement tools to measure the height and weight of the infants and young children in the study. The tools underwent standard calibration procedures prior to use. Infants and young children were required to remove their shoes and hats, and wear light clothing for the measurements. Height was measured in centimeters, with an accuracy of 0.1 cm. Upper arm circumference was measured in centimeters, with an accuracy of 0.1 cm, and weight was measured in kilograms, with an accuracy of 0.1 kg. The diagnostic criteria for mild malnutrition in infants and young children included z‐scores calculated using Anthro software. Diagnoses were based on the recommended ranges for pediatric nutritional assessments published by AND and ASPEN in 2014 [6]. The diagnostic criteria require meeting any one of the following criteria (while excluding moderate or severe malnutrition): a weight‐for‐height z‐score between −2 and −1, a BMI‐for‐age z‐score between −2 and −1, and an MUAC z‐score between −2 and −1 [6].

#### 2.2.2 Questionnaire Survey

A self‐designed questionnaire survey was conducted on the day of the infant's physical examination. Surveyors interviewed the primary caregiver of the infant face‐to‐face to collect information on the infant's general condition, family and living environment, feeding status, sleep type, food allergies, maternal pre‐pregnancy nutritional status, and mother's weight gain during pregnancy.

#### 2.2.3 Variable Grouping and Definition

The definitions for variables are listed below.

Insufficient sleep: The National Sleep Foundation recommends sleep duration of < 12 h for infants aged 6–12 months and < 11 h for young children aged 12–24 months.

Frequent nighttime awakenings: According to the National Sleep Foundation [12], one nighttime awakening is considered to be a sleep interruption lasting > 5 min that requires soothing or feeding to resume sleep; > 2 awakenings per night is considered frequent nighttime awakenings.

Sleep chronotype: Earlier chronotype sleep time is concentrated between 8 p.m. and 4 a.m. Later chronotype sleep time is concentrated between 4:00 a.m. and 12:00 p.m.

Insufficient complementary feeding frequency: The recommended complementary feeding frequency in the Guidelines for Infant and Toddler Nutritional Feeding Assessment Services issued by the General Office of the National Health Commission of China is 1–2 times per day for infants aged 6–8 months and 2–3 times per day for infants aged 9–12 months. Three times per day is considered adequate for those aged 12–24 months.

Food allergy: The gold standard for diagnosing a food allergy is the food elimination–challenge test; however, this diagnostic method is difficult to implement due to low compliance from the child's family. In this study, food allergy diagnoses were primarily based on the medical history, a family history investigation, and physical examination results, combined with serum‐specific immunoglobulin E (IgE) testing, allergen skin prick tests, and total blood eosinophil counts for comprehensive evaluation.

### 2.3 Sample Size Calculation

The sample size for this study was calculated based on a cross‐sectional survey design. The sample size was estimated using the population prevalence estimation method. The corresponding formula is given as follows:

$$n=\frac{z_{\sigma }^{2}\times p\;\left(1-p\right)\;}{d^{2}},$$

where *n* represents the theoretical minimum sample size, *z*_*σ*_ denotes the significance test statistic, with *α* = 0.05 yielding *z*_*σ*_ = 1.96, and *d* signifying the margin of error, set at 0.2 *p*. Preliminary surveys indicated a prevalence rate (*p*) of approximately 13% for mild malnutrition among infants aged 6–24 months. Thus, the theoretical minimum sample size was calculated to be 643 cases. Considering a potential 20% nonresponse rate in the study survey, the final required sample size was 771 cases.

### 2.4 Statistical Methods

After collecting the questionnaires, two investigators checked them and entered the data into Excel 2019 software. SPSS 27.0 software was used to analyze the data. Count data were described as the number of cases (%). Univariable analysis was performed using the chi‐squared (*χ* ^*2*^) test, with *p* values of < 0.05 indicating statistically significant differences. Variables with statistical significance in the univariable analysis were used as independent variables, and infantile mild malnutrition was used as the dependent variable in the multivariable analysis. Multivariable analysis was performed using bivariate logistic regression analysis. The significance level was set at *α* = 0.05 (two‐tailed).

## 3 Results

### 3.1 Basic Information

The study examined a total of 879 infants and young children aged 6–24 months. Among them, 63 male infants (13.5%, 63/465) and 59 female infants (14.3%, 59/414) were diagnosed with mild malnutrition. The detection rates of mild malnutrition between the sexes were not significantly different (*P* = 0.764). Among infants aged 6 to < 9 months, 38 cases of mild malnutrition were identified, yielding a detection rate of 13.8% (38/275). Twenty‐six cases were identified among those aged 9 to < 12 months, for a detection rate of 13.9% (26/187). Among infants aged 12 to < 18 months, 33 cases of mild malnutrition were detected, for a detection rate of 14.7% (33/224), and 25 cases of mild malnutrition were detected among infants aged 18 to < 24 months, yielding a detection rate of 13.0% (25/193). The detection rates of mild malnutrition across different age groups were not significantly different (*P* = 0.965) (Table 1).

**TABLE 1:** Distribution of mild malnutrition by gender and screening age.

Variable	Children with mild malnutrition, *n* (%)	Children without malnutrition, *n* (%)	Total children, *n*	*p* value
Sex				0.764
Boys	63 (13.5)	402 (86.5)	465	
Girls	59 (14.3)	355 (85.7)	414	
Age group				0.965
6–< 9 months	38 (13.8)	237 (86.2)	275	
9–< 12 months	26 (13.9)	161 (86.1)	187	
12–< 18 months	33 (14.7)	191 (85.3)	224	
18–< 24 months	25 (13.0)	168 (87.0)	193	

### 3.2 Univariable Analysis of Mild Malnutrition

A univariable analysis was conducted on the nutritional status of 879 infants aged 6–24 months, whose complete questionnaires were obtained. The results showed statistically significant differences in the prevalence of mild malnutrition according to complementary feeding frequency, food allergies, maternal prepregnancy nutritional status, maternal education level, monthly household income per capita, total sleep duration, sleep chronotype, nighttime awakenings, and low birth weight (*p* < 0.05) (Table 2).

**TABLE 2:** Univariate analysis of factors affecting mild malnutrition.

Variable	Children with mild malnutrition, *n* (%)	Children without malnutrition, *n* (%)	*p* value
Complementary feeding frequency			
Sufficient	65 (11.5)	499 (88.5)	0.007
Insufficient	57 (18.1)	258 (81.9)	
Food allergy			
Yes	34 (30.9)	76 (69.1)	< 0.001
No	88 (11.4)	681 (88.6)	
Maternal pre‐pregnancy nutritional status			
BMI < 18.5 kg/m^2^	23 (26.1)	65 (73.9)	< 0.001
BMI ≥ 18.5 kg/m^2^	99 (12.5)	692 (87.5)	
Maternal education			
High school and below	27 (23.3)	89 (76.7)	0.002
College degree or above	95 (12.5)	668 (87.5)	
Monthly household income per capita			
< 5000 Yuan	47 (19.3)	197 (80.7)	0.004
≥ 5000 Yuan	75 (11.8)	560 (88.2)	
Total sleep duration			
Sufficient	106 (13.1)	704 (86.9)	0.020
Insufficient	16 (23.2)	53 (76.8)	
Sleep chronotype			
Earlier chronotypes	105 (13.0)	700 (87.0)	0.018
Later chronotypes	17 (23.0)	57 (77.0)	
Nighttime awakenings			
Frequent	32 (27.1)	86 (72.9)	< 0.001
Not frequent	90 (11.8)	671 (88.2)	
Birth weight			
< 2500 g	26 (22.4)	90 (77.6)	0.004
≥ 2500 g	96 (12.6)	667 (87.4)	

Abbreviation: BMI, body mass index.

### 3.3 Multivariable Logistic Regression Analysis of Mild Malnutrition

Statistically significant variables (*p* < 0.05) in the univariable analysis were subjected to variance inflation factor (VIF) testing to avoid severe multicollinearity issues among variables. The results showed that all variables had VIF values below 5, indicating no multicollinearity problems among them. Mild malnutrition was used as the dependent variable, and the following independent variables with statistical significance in the univariable analysis were included in the multivariate logistic regression analysis: low birth weight, insufficient complementary feeding frequency, food allergies, maternal educational levels, monthly household per capita income less than 5000 Chinese yuan, maternal pre‐pregnancy thinness, insufficient total sleep, inverted sleep patterns, and frequent nighttime awakenings. The results showed that food allergies (odds ratio (OR) = 3.117, 95% confidence interval (CI): 1.902–5.108), low maternal education level (OR = 1.969, 95% CI: 1.166–3.325), low birth weight (OR = 1.759, 95% CI: 1.041–2.973), maternal pre‐pregnancy BMI < 18.5 kg/m^2^ (OR = 2.197, 95% CI: 1.252–3.855), frequent nighttime awakenings (OR = 2.427, 95% CI: 1.481–3.975), and insufficient complementary feeding frequency (OR = 1.550, 95% CI: 1.030–2.332) were identified as risk factors for mild malnutrition in infants aged 6–24 months (Table 3).

**TABLE 3:** Univariate analysis of factors affecting mild malnutrition.

Variable	β	SE	World *χ²*	*p* value	OR	95% CI
Insufficient complementary feeding frequency	0.438	0.209	4.410	0.036	1.550	1.030–2.332
Food allergy	1.137	0.252	20.351	< 0.001	3.117	1.902–5.108
Maternal pre‐pregnancy BMI < 18.5	0.787	0.287	7.525	0.006	2.197	1.252–3.855
Low maternal education level	0.678	0.267	6.429	0.011	1.969	1.166–3.325
Monthly household income per capita < 5000 Yuan	0.394	0.221	3.166	0.075	1.482	0.961–2.287
Insufficient total sleep duration	0.522	0.335	2.429	0.119	1.685	0.874–3.250
Later chronotypes	0.418	0.325	1.655	0.198	1.520	0.803–2.875
Frequent nighttime awakenings	0.887	0.252	12.397	< 0.001	2.427	1.481–3.975
Low birth weight	0.565	0.268	4.455	0.035	1.759	1.041–2.973

Abbreviations: BMI, body mass index; CI, confidence interval; OR, odds ratio.

## 4 Discussion

The detection rate of mild malnutrition among 879 infants and toddlers in the Outpatient Clinic of Pediatric Health Care at The Third Affiliated Hospital of Zhengzhou University during the period from December 2024 to August 2025 was 13.88%. Univariable analysis revealed that factors such as low birth weight, the frequency of complementary feeding, food allergies, maternal educational level, household monthly income, maternal prepregnancy underweight, insufficient total sleep duration, sleep chronotype, and frequent night awakenings were associated with mild malnutrition in infants and young children aged 6–24 months. Further multivariable analysis indicated that low birth weight, food allergies, maternal education level, maternal prepregnancy thinness, frequent nighttime awakenings, and infrequent complementary feeding were significant factors influencing mild malnutrition in this age group.

Food allergies mainly occur in early life. This could be related to the immaturity of organ systems, especially the immune system, during this period. The immune response pattern in early life is dominated by Th2 cell‐mediated immune reactions. This immune phenotype is highly correlated with the mechanism of immediate allergic reactions, providing an immunological basis for the high incidence of food allergies in infants and young children [13]. The incidence of food allergies has increased worldwide in recent years [14]. These increases may be related to environmental factors, changes in feeding patterns, advances in clinical diagnostic techniques, and a significant increase in the public awareness of allergies. The association between food allergies and the nutritional status of infants and young children has long been a subject of debate. The results of this study indicate that food allergies are a risk factor for mild malnutrition in infants and young children, consistent with the conclusions of previous studies suggesting that food allergies may interfere with nutrient metabolism. [15, 16, 17]. In an allergic state, the immune system is activated, resulting in an increased basal metabolic rate and a significant increase in the demand for nutrients. The local inflammatory response in the intestines triggered by allergies can damage intestinal mucosal barrier function [17], leading to increased intestinal permeability and impaired nutrient absorption. This process may also disrupt the intestinal microbiota balance, further exacerbating nutritional metabolic disorders. Multiple food allergies and excessive dietary restrictions imposed by some parents can significantly narrow the range of food options available to infants and young children [18, 19]. These factors interact synergistically, ultimately exerting a significant negative impact on the nutritional status of infants and young children.

The family is the most fundamental social environment for children's growth and survival, with mothers typically serving as the primary caregivers and educators within the family. A study has indicated a positive correlation between a mother's educational attainment and children's nutrition [20]. Higher educational attainment among mothers is associated with greater utilization of healthcare services, better ability to navigate the healthcare system, higher levels of parenting knowledge and skills, and openness to new ideas, all of which contribute to more effective prevention and improvement of infant and toddler malnutrition. Highly educated mothers also tend to have greater decision‐making authority and better resource allocation capabilities during the early years of a child's development [21]. Thus, mothers are more likely to prioritize resource allocation for infants, thereby reducing the risk of growth deficiencies [22].

Low birth weight is defined as a birth weight of less than 2500 g, typically indicating preterm birth or intrauterine growth restriction [23]. The subsequent increase in height and weight is closely related to early infant nutrition. Studies indicate that low birth weight infants experience sustained effects on both early‐life and long‐term physical development [24, 25]. Infants with low birth weight exhibit an “accelerated catch‐up” pattern of growth and development during the early stages of life, particularly during the catch‐up growth phase. Compared to full‐term, term‐appropriate infants, they have greater requirements for energy, protein, and minerals. However, infants with low birth weight, especially preterm infants, often have impaired digestive and absorptive functions, as well as poor coordination of sucking and swallowing, making them more prone to nutritional issues.

Nutritional programming during early life has profound implications for individual health, and the mother's nutritional status plays a critical role in this process. Prepregnancy underweight status directly leads to low birth weight in infants and has far‐reaching effects on long‐term physical development [26, 27, 28, 29]. Animal model studies have revealed that protein restriction from preconception to lactation induces epigenetic modifications in ribosomal DNA (rDNA). These DNA methylation changes persist into adulthood and directly lead to growth‐restricted phenotypes [30]. This suggests that preconception malnutrition can permanently impair infants' growth potential. The maternal nutritional and metabolic status prior to pregnancy has a more significant impact on offspring growth and development than prenatal nutrition [28]. The adverse intrauterine environment caused by preconception malnutrition can program the metabolic and immune functions of offspring, both increasing the incidence of mild malnutrition during childhood and also potentially elevating the risk of chronic diseases in adulthood, creating intergenerational health risks [31].

Sleep, as a core physiological activity that sustains the brain's vitality, is not only a key process through which the body conserves and restores energy but also holds special significance for infants and young children. Infant sleep patterns develop rapidly by 3 months of age and are largely established by 6 months [32]. As infants grow older, their sleep patterns gradually stabilize and are characterized by longer nighttime sleep and shorter daytime sleep. Infants and young children require significantly more sleep than adults. Adequate and high‐quality sleep is essential for their physical growth and also plays a crucial role in the development of the immune system, the refinement of cognitive functions, emotional regulation [33], and memory capacity. Our study has demonstrated that frequent nighttime awakenings correlates with mild malnutrition. Frequent nighttime awakenings can negatively impact the current body weight, height, and chest circumference of infants and toddlers [34]. The mechanism may involve the disruption of slow‐wave sleep by these awakenings. Human growth hormone (GH) exhibits pulsatile secretion that is highly coupled with the sleep‐wake cycle, with GH release peaking after slow‐wave sleep onset. This association is highly stable [35, 36]. A study indicates that reduced slow‐wave sleep is significantly associated with short stature in prepubescent children [37]. GH directly stimulates epiphyseal plates and indirectly promotes growth by inducing insulin‐like growth factor‐1 production, which facilitates the endochondral ossification of long bones [38]. Frequent nighttime awakenings may significantly reduce or eliminate high‐amplitude GH pulses. Ultimately, this results in delayed increases in height, weight, and other physical indicators. A study has indicated that introducing solid foods or complementary foods early may be associated with decreases in nighttime awakenings. This suggests that frequent nighttime awakenings may be related to insufficient food intake density [39]. Infants have limited stomach capacity, and solid foods have significantly higher energy and nutrient density per unit volume than breast milk or formula. Thus, they meet the physiological needs of infants more effectively over an extended period.

After 4–6 months of age, infants and young children enter a new stage of growth and development. Feeding only breast milk or formula can insufficiently meet the nutritional needs for key nutrients such as iron, copper, zinc, and vitamin B6 [40, 41, 42]. The World Health Organization recommends introducing complementary foods starting at 6 months of age [43]. The period from 6 to 24 months represents the core window for transitioning infants from exclusive milk feeding to regular family meals. During this phase, complementary foods gradually supplement and replace breast milk or formula, providing essential nutrients for the developing digestive system while regulating microbial colonization [44, 45]. Achieving adequate complementary feeding frequency is a core indicator for assessing feeding practices. Given infants' small stomach capacity and limited portion sizes per meal, ensuring frequent feedings is crucial to match energy and nutrient intake with rapid growth. Insufficient stimulation from complementary foods prevents the establishment of stable beneficial gut microbiota, reducing nutrient absorption and leading to hidden malnutrition where nutrients are ingested but not absorbed.

This study has the following limitations. First, the research data were obtained from a single center, and the sample size was relatively small, which limits the representativeness of the study results, reflecting only the current situation of mild malnutrition among infants and young children aged 6–24 months in urban Zhengzhou. Cross‐sectional studies struggle to dynamically observe the temporal relationships between variables, and some questions in the questionnaire survey regarding past feeding behaviors and health status may be subject to recall bias due to unclear memories of the respondents, thereby affecting the accuracy of the results.

Second, this study did not employ food challenge tests (the gold standard) for diagnosing food allergies. Instead, diagnoses were made through a comprehensive assessment of medical history, specific IgE testing, and a physical examination. Although this diagnostic approach aligns with clinical practice, it carries a certain risk of misdiagnosis or missed diagnosis. This may slightly affect the accuracy of the analysis of the association between allergies and malnutrition. Future studies should incorporate the gold standard to enhance diagnostic precision. Finally, this study excluded children with moderate‐to‐severe malnutrition to minimize confounding effects from severe pathological conditions. This design limitation may restrict the generalizability of the findings to broader pediatric populations. Future research should encompass the full spectrum of malnutrition severity to validate the universality of the identified risk factors across varying nutritional statuses.

## 5 Conclusions

Mild malnutrition is the initial stage of nutritional deficiency. Early identification and systematic intervention for infants and young children with mild malnutrition are critical measures to prevent the progression of malnutrition and reduce the incidence of moderate‐to‐severe malnutrition. This study identified low birth weight, food allergies, maternal education level, maternal pre‐pregnancy thinness, frequent nighttime awakenings, and insufficient complementary feeding frequency as risk factors for mild malnutrition in infants and young children aged 6–24 months. Based on these findings, clinical practice should strengthen systematic nutrition and health guidance for women during the periconceptional period. Periconceptional nutrition management should be integrated as a core component of preconception care and prenatal checkups. Concurrently, the caregivers of infants and young children should be educated on scientific feeding practices and the importance of sleep hygiene for infants and young children. Furthermore, individualized nutritional management should be prioritized for children with allergies. Nutritional follow‐up records should be established for allergic children, with pediatric nutritionists developing alternative nutrient supplementation plans. This ensures the adequate intake of core nutrients, including high‐quality protein, iron, and vitamins, while avoiding allergic risks and preventing nutritional gaps caused by food restrictions. By constructing a comprehensive, multidimensional infant and toddler nutritional health intervention system, we aim to precisely prevent and control the risk of mild malnutrition in infants and young children.

## Author Contributions

Li Song and Qian Li reviewed and revised the manuscript. Menghua Yin performed the data analysis, drafted the initial manuscript, and revised it. Menghua Yin, Rumeng Yu, Mengxuan Zhou, and Wenxiu Wu collected the data. All authors approved the final version of the manuscript and agreed to its publication.

## Ethics Statement

This study was approved by the Ethics Committee of The Third Affiliated Hospital of Zhengzhou University (ethics approval number: 2024‐428‐01). The informed consents were obtained from the family members.

## Conflicts of Interest

The authors declare no conflicts of interest.

## Data Availability

The datasets of this study are not publicly available due to patient privacy concerns, but can be obtained from the corresponding author upon reasonable request.
